# 2025 position statement on active outdoor play: process and methodology

**DOI:** 10.1186/s12966-025-01806-8

**Published:** 2025-09-25

**Authors:** Louise de Lannoy, Eun-Young Lee, Suryeon Ryu, Olivia Lopes, Joshua S. Cheruvathur, Anujah Thankarajah, Dina Adjei Boadi, Isabel de Barros, Scott Duncan, Maeghan E. James, Lærke Mygind, Robyn Monro Miller, Leigh M. Vanderloo, Po-Yu Wang, Laura Heather, Mark S. Tremblay

**Affiliations:** 1 Outdoor Play Canada, Ottawa, Canada; 2 Queen’s University, Kingston, Canada; 3 Children’s Hospital of Eastern Ontario Research Institute, Ottawa, Canada; 4 Department of Sport Industry, Yonsei University, Seoul, South Korea; 5 University of Tennessee, Knoxville, USA; 6 University of Ottawa, Ottawa, Canada; 7 University of Guelph, Guelph, Canada; 8 University of Ghana, Accra, Ghana; 9 Instituto Alana, São Paulo, Brazil; 10 Auckland University of Technology, Auckland, New Zealand; 11 Copenhagen University Hospital, Copenhagen, Denmark; 12 International Play Association, Brisbane, Australia; 13 ParticipACTION, Toronto, Canada; 14 University of Western Ontario, London, Canada; 15 National Taiwan Normal University, Taipei, Taiwan; 16 Lakehead University, Thunder Bay, Canada; 17 Carleton University, Ottawa, Canada

**Keywords:** Active outdoor play, Health and wellbeing, Education and learning, Movement behaviors, One health, Nature and environment, Human rights and policy, Community connections, Social capital

## Abstract

**Background:**

The 2015 Position Statement on Active Outdoor Play highlighted the benefits of active outdoor play for child health and development and had significant impact on public policy, law, philanthropy, and research in Canada and internationally. To celebrate its 10th anniversary, we led an update to the Position Statement that is global in scope and inclusive of all human age groups. The goal of this manuscript is to outline the process and methodology involved in developing the 2025 Position Statement on Active Outdoor Play.

**Methods:**

The process involved several key steps: (1) establishing an international leadership group; (2) forming an international steering committee; (3) conducting an environmental scan of historical key events in active outdoor play; (4) using text-mining and linked network analyses to identify core research themes and map relationships among outdoor play organizations; (5) developing a conceptual framework through a consensus-building process; and, (6) using this conceptual framework to guide the completion of a series of reviews used to inform the 2025 Position Statement.

**Results:**

The environmental scan identified 136 key events that provided historical context for the evolution of active outdoor play. Text-mining and linked network analyses revealed 10 core research themes and mapped relationships among key organizations and individuals in the field. Informed by these themes and concepts, a draft conceptual framework was developed and circulated to the > 130-person steering committee for review and consensus. The final version of the conceptual framework encompassed 9 key themes, which guided the focus of 18 reviews used to inform the scope and content of the 2025 Position Statement on Active Outdoor Play.

**Conclusions:**

A three-year-long process involving an international leadership group and >130 person steering committee led to the development of an environmental scan, text-mining and linked network analysis, and a conceptual framework including 9 key themes with bidirectional relationships with active outdoor play that guided the development of 18 reviews. Collectively, this work served to inform the updated Position Statement on Active Outdoor Play, which aims to encourage humans of all ages to play, learn, teach, grow, and thrive outdoors and serve as caring stewards of the land, water, air, plants, animals, and each other.

**Supplementary Information:**

The online version contains supplementary material available at 10.1186/s12966-025-01806-8.

## Background

The 2015 Position Statement on Active Outdoor Play [1] and its supporting evidence [2, 3] highlighted the benefits of active outdoor play for children’s growth, development, and mental, emotional, cognitive, and social health. The 2015 Position Statement provided the outdoor play sector in Canada with both direction and a common purpose: to promote healthy growth and development among children in harmony with the outdoors and the natural environment. The 2015 Position Statement also served as a galvanizing force for the outdoor play sector in Canada, bringing together previously disconnected groups from education, community, health, environment, wildlife, ecology, law, and Indigenous rights that collectively shared a passion for outdoor play. Its impact has been far-reaching and prompted the Lawson Foundation to invest ~$13 M in Canadian projects focused on increasing children’s opportunities for outdoor play [4]. The Position Statement also informed a British Columbia Supreme Court decision against a lawsuit demonizing outdoor play [5]. It led to a tenfold increase in outdoor play research publications in Canada [6, 7] and inspired the official launch of Outdoor Play Canada (OPC) [8]a national organization dedicated to leading, connecting, and amplifying the work of Canada’s outdoor play community. The Position Statement’s influence extended internationally, in part through the establishment of the Play, Learn, and Teach Outdoors Network (PLaTO-Net) [9] an international initiative housed in OPC that now has > 700 members from > 60 countries. Additionally, it spurred the development of the *Outdoor Play in Canada: 2021 State of the Sector Report* [10] which served as a reflective strategic documentation of the first five years following the release of the 2015 Position Statement. This report reviewed the state of outdoor play during the global pandemic and identified nine priorities to further advance the outdoor play sector in Canada. The 2015 Position Statement continues to serve as a foundational document, guiding local, provincial, national and international efforts to promote outdoor play as an essential component of children’s health, well-being, and connection to the natural world.

In 2025, it will be 10 years since the release of the Position Statement on Active Outdoor Play. In celebration of the many projects, initiatives, and other successes it has inspired in the past decade, OPC assembled an international 11-person leadership group consisting of researchers and thought leaders representing every inhabited continent to spearhead the project—and a larger steering committee consisting of over 130 researchers, practitioners, policy-makers, and other active outdoor play-relevant actors and groups to guide and provide input on the development of the 2025 Position Statement of Active Outdoor Play (hereafter called the ’AOP10’ project). Given the number of new projects and initiatives that have emerged since the 2015 Position Statement release, and increasing recognition of the value of outdoor play for all ages [6], the AOP10 project provides an opportunity to describe its impact, consolidate similar Position Statements – such as those of the Council of Chief Medical Officers of Health [11] and the Canadian Pediatric Society [12]—and broaden the network of stakeholders including those invested in outdoor play across all age groups. Furthermore, given that the 2015 Position Statement has gained international recognition and influence, partly through the establishment of PLaTO-Net, this project is, therefore, an opportunity to extend its global reach, scope and relevance [13]. 

As highlighted in the World Health Organization’s (WHO) Global Action Plan on Physical Activity 2018–2030 [13], promoting opportunities for active play may help achieve at least 13/17 of the United Nation’s Sustainable Development Goals (SDGs)—for example, SDG3 (Good Health and Well-being), SDG11 (Sustainable Cities and Communities), and SDG15 (Life on Land) [14]—emphasizing the cross-sectoral nature and benefit of play across all ages. Accordingly, the AOP10 project is age-inclusive and examines evidence with a life-course perspective. The AOP10 project serves as the opportunity to highlight key learnings from the past decade and explore how these insights can be leveraged for multifaceted, scalable, and sustainable benefit in promoting active outdoor play and health globally and for all ages.

The AOP10 project involved five sequential stages:

1. Creation of a leadership structure to guide the AOP10 project and the development of a conceptual framework to establish the project scope and identify important topics/themes to be covered in the updated Position Statement.
2. Assess the current state of evidence by conducting a series of reviews on the themes identified in the conceptual framework.
3. Create an initial draft of the 2025 Position Statement by aggregating and synthesizing the findings from stages 1 and 2 above.
4. Achieve consensus on the updated Position Statement by sharing interactive drafts with stakeholders through a series of surveys, similar to what was done for the 2015 Position Statement.
5. Share the work broadly by publishing the research reviews and final *2025 Position Statement on Active Outdoor Play*, presenting this work at events/meetings in Canada and internationally, and creating digital summaries, slide decks for broad use, infographics, interviews, and curated social media content. To ensure global reach, the 2025 Position Statement will be available (at minimum) in the six United Nations official languages (Arabic, Chinese, English, French, Russian, Spanish).

This manuscript outlines the first two stages; a robust process that resulted in twelve systematic reviews and six narrative reviews led by our international leadership group and steering committee that informed the development of the initial AOP10 draft. A separate manuscript is being developed for the final *2025 Position Statement on Active Outdoor Play*. The decision to separate this large project into two papers was two-fold: first, given the robustness of our methodology, dividing this project into a methods paper and final Position Statement paper allowed for the development of two more manageably sized manuscripts; and second, the process involved in creating this update is novel in its scope and international reach. The purpose of this paper was therefore to describe the robust process that resulted in twelve systematic reviews and six narrative reviews led by our international leadership group and steering committee that informed the development of the initial AOP10 draft.

## Methods

## Establishing leadership and developing a conceptual framework for the AOP10 project

### AOP10 leadership group

**Fig. 1:**
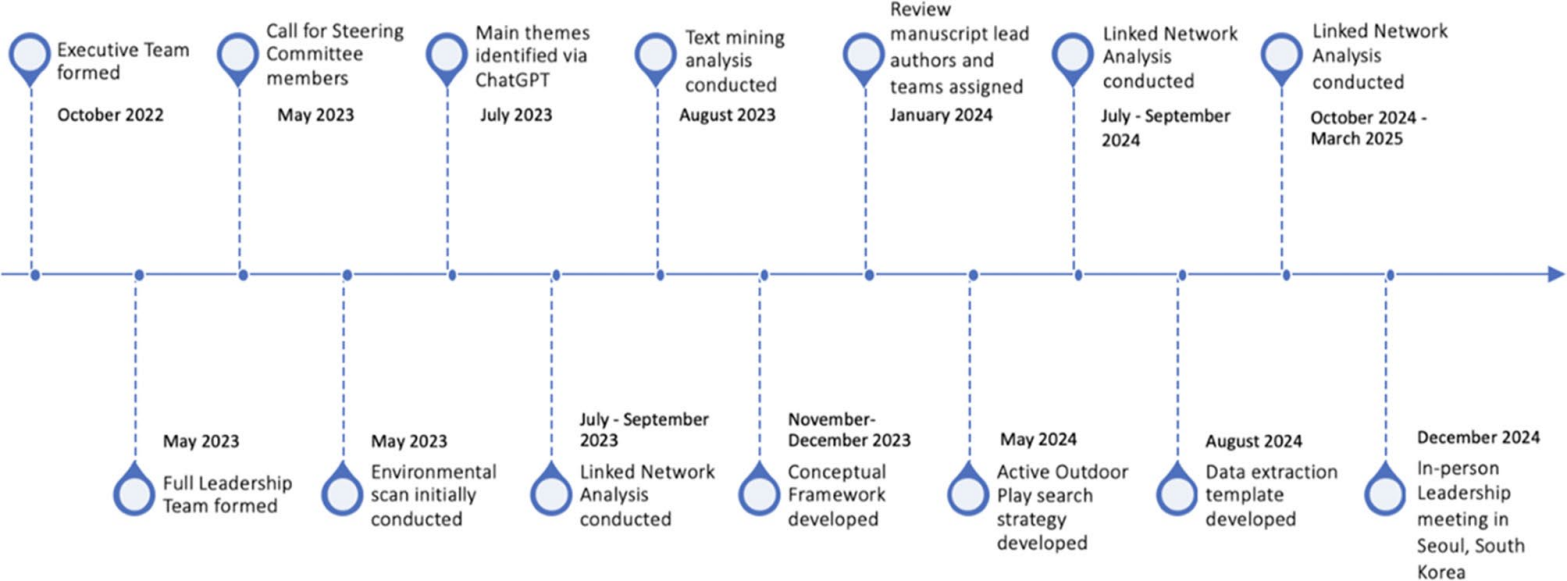
Flowchart depicting the timeline of the first stage of the AOP10 project

An executive team for this project, consisting of the chair and co-founder of the Play, Learn and Teach Outdoors Network (E-YL; PLaTO-Net), the president of OPC (MST) and the executive director of OPC (LDL), was initially formed in October 2022 (see Fig. 1 timeline). This executive team used their established outdoor play networks to create a larger leadership group to include outdoor play experts from every inhabited continent. One trainee from the Children’s Hospital of Eastern Ontario Research Institute and two additional leadership group members from a Canadian national thought leadership organization (ParticipACTION) and an international play organization (International Play Association) were added to ensure broad reach and dissemination capacity. A list of the leadership group members, home country, and affiliation are provided in Supplemental File 1. The full AOP10 leadership group began meeting monthly in May 2023 to discuss and lead the scope, process, and parameters of the project. At the start of the project, the leadership group agreed that the 2025 Position Statement on Active Outdoor Play be inclusive of all ages (i.e., children, adolescents, adults, and older adults), in alignment with the definition of active outdoor play from the PLaTO-Net project: *Voluntary engagement in activity that is fun and/or rewarding and usually driven by intrinsic motivation*,* that takes place outdoors and involves physical activity of any intensity* [15]. 

### Steering committee

Following the formation of the AOP10 leadership group, a call was put out on the OPC website inviting outdoor play leaders and relevant actors and groups to join this project by submitting an expression of interest (EOI) of no more than 500 words to the general OPC email (info@outdoorplaycanada.ca). Targeted invitations were sent to those involved in adult-focused outdoor play and recreation to ensure relevant scholars and experts in this realm were represented. All steering committee submissions outlining an individual’s interest in and relevant background experience for this project, were accepted. This call was further promoted on social media (Instagram, Twitter/X, Facebook) and at two international conferences (the 2023 International Play Association Conference in Glasgow, Scotland and the 2023 International Society for Behavioral Nutrition and Physical Activity Conference in Uppsala, Sweden) by the leadership group.

To ensure global experts within the outdoor play sector were aware of, and had opportunity to participate in this call, the leadership group sent an email to the full PLaTO-Net membership (622 members from over 50 countries at the time) seeking their interest in participating in the project (EOIs were again requested and required to join). In addition, Google Scholar was searched to identify leading researchers within the fields of outdoor play, risky play, play (with further investigation into whether the identified experts were involved in outdoor-focused play research), outdoor learning, and outdoor teaching and pedagogy; the top five individuals in each field were identified and then contacted directly by the executive team seeking their participation. A list of the final steering committee members, home country, and affiliation are provided in Supplemental File 1.

### Project scope

The scope of the Position Statement update was determined through (1) evidence from an environmental scan, (2) the use of ChatGPT-4 (Chat Generative Pre-Trained Transformer-4), a large language model (LLM), to identify common themes across previously published Position Statements related to active outdoor play, (3) text-mining methods to identify hot and cold topics in the area of active outdoor play, and (4) a linked network analysis to gain a deeper understanding of the structure and function of a complex system—in this case, the relationships among cross-sectoral active outdoor play leaders in Canada. Based on these efforts, central themes and subthemes were compiled by LDL and OL which were then reviewed by the executive team, followed by the leadership group, who collectively developed a conceptual framework encapsulating these themes and subthemes. A survey was then distributed to all steering committee members (*n* = 105 at the time) to assess the level of agreement on the draft conceptual framework and provide an opportunity for additional feedback. The detailed processes of developing this project scope are described in the following sections.

#### Data collection for project scope - environmental scan

As a preliminary step to developing the project scope and subsequent conceptual framework, a global environmental scan of the history and development of outdoor play was conducted to understand how this area evolved into the sector it is today. All items included in this scan were large-scale events (international or national) that contributed to the development and/or progression of the outdoor play sector.

As there is no single established method for conducting an environmental scan the “formal searching” method first outlined by Choo in 2001 [16] was adopted. This method has been used by our group and others in recently published environmental scans [17–19]. The search consisted of three main parts: (i) scan of resources on the OPC website; (ii) grey literature and data search of Google; and (iii) key informant consultations. The detailed processes of this environmental scan are described in the following sections.

##### Environmental scan - scan of the Outdoor Play Canada website

The OPC website hosts a large database of credible resources and information on the outdoor play sector in Canada and beyond, serving as the launching point for this environmental scan. The ‘Resources’ database and timeline in the ‘About’ page under ‘Our Story’ consisted of 220 items that were screened. In some instances, resources led to the identification of additional items relevant to our search and were therefore investigated further. The OPC website was particularly useful in identifying major events that initiated the global outdoor play movement in the 20th century.

##### Environmental scan - grey literature and data search on Google

A grey literature and data search was conducted on Google from May to June 2023 using the terms and parameters identified below. The following types of resources were extracted from our search: conferences, seminars, summits, fact sheets, position statements, declarations, projects, contests, outdoor play-related holidays, and formations of major outdoor play organizations, inclusive of all ages. In accordance with the approach previously used by Jenkins and colleagues [18], the first 20 links generated on Google were screened for relevance. When a website relevant to outdoor play was identified, the next 10 links were reviewed until no additional relevant links were identified. Additionally, any relevant links within a website identified through this search process were investigated until the content became irrelevant to the search.

The following keywords were used while conducting the grey literature and data search on Google:

international, national, outdoor play, play, events, developments, Europe, South America, North America, Asia, Africa, Oceania, children*(’s), and right*(s). These keywords were used in combination as a search strategy to find relevant items for the environmental scan. Efforts were made to limit the impact of search location on the results by turning off the researcher’s location when conducting Google searches and using a private web browser.

##### Environmental scan - key informant consultations

The environmental scan was conducted as one of the preliminary steps for the AOP10 project. To ensure international outdoor play efforts were well represented in this scan, leaders of this project reached out to former steering committee members of the PLaTO-Net terminology, taxonomy, and ontology international consensus project (*n* = 22),^20^ seeking their knowledge of organizations/groups focused on promoting active outdoor play inclusive of all ages in their country/jurisdiction. The list derived from this network effort was cross-referenced with the resources and organizations identified through other scan efforts as listed above (Supplemental File 2).

#### Data collection for project scope – ChatGPT

We compiled all position statements related to active outdoor play and learning (documented on the OPC website, shared through author networks, or gathered through the environmental scan), then requested ChatGPT to identify the overarching themes among all position statements (see Table 1 in Results).

Similarly, we compiled all EOIs from steering committee members, then requested ChatGPT to identify the overarching themes among all EOIs (Table 1; Results). Due to the character limit of ChatGPT, we used ChatGPT to identify shared themes in groups of 10 EOIs prior to identifying the overarching themes across all EOIs.

A similar process was conducted to identify the common areas of expertise and areas of interest across all EOIs (Table 1; Results). We requested ChatGPT to identify the top areas of expertise/interest of each EOI based on their full text, and the overarching areas among all top areas of expertise/interest of each EOI. Again, due to the character limitation of ChatGPT, we used ChatGPT to identify the shared areas of expertise/interest in groups of 10 EOIs prior to identifying the overarching areas of expertise/interest across all EOIs (Supplemental File 3). All prompts were developed by the AOP10 executive team and independently submitted by a student. We made every effort to use prompts that did not involve overconfident, biased language and we additionally integrated external knowledge to inform the model response, collectively reducing the risk of sycophancy [20]. 

#### Data collection for project scope – text mining

Text mining was leveraged to identify and investigate the research patterns within the field of active outdoor play. The specific research question was: W*hat are the 10 key research topics in active outdoor play literature*?

##### Text mining – process

Text data—including the publication year, title, and abstract—were retrieved from bibliographic records using Web of Science. The search keywords were determined based on discussions among the executive team. After screening for records with missing abstracts, SR with the support of E-YL conducted the text-mining analyses, which included pre-processing and Latent Dirichlet Allocation (LDA) topic modeling techniques [21]. Finally, the executive team confirmed the appropriate subtitles for each topic entity.

##### Text mining – data collection

The data used for text-mining analyses (i.e., topic modeling techniques) were obtained from the Web of Science database on August 16, 2023. The advanced search was performed in the search field tag labeled “topic,” using keywords related to the following topics: (1) active outdoor play; (2) One Health [22]; (3) climate change; (4) mental health; (5) environmental stewardship; (6) crime; (7) overall health and well-being; (8) cultural appreciation and global understanding; (9) equity, diversity, and inclusion (EDI) and, (10) parenting. The search was conducted using the following combination of keywords and Boolean operator strings: #1 AND (#2 OR #3 OR #4 OR #5 OR #6 OR #7 OR #8 OR #9 OR #10 OR #11). The specific combinations of keywords and Boolean operator strings are shown in Supplemental File 4. In terms of the eligibility criteria for included documents, the results were refined to include only those in the Science Citation Index Expanded and Social Sciences Citation Index and were limited to specific document types (i.e., articles, proceedings, review articles, early access, editorial material), language (English), and publication years (from 2000-01-01 to 2023-08-01). The research included 7,719 documents and a total of 7,696 documents were used for the analyses; 23 documents were excluded as they did not have an abstract (17) or were duplicate articles (6).

##### Text mining – data processing

**Fig. 2:**
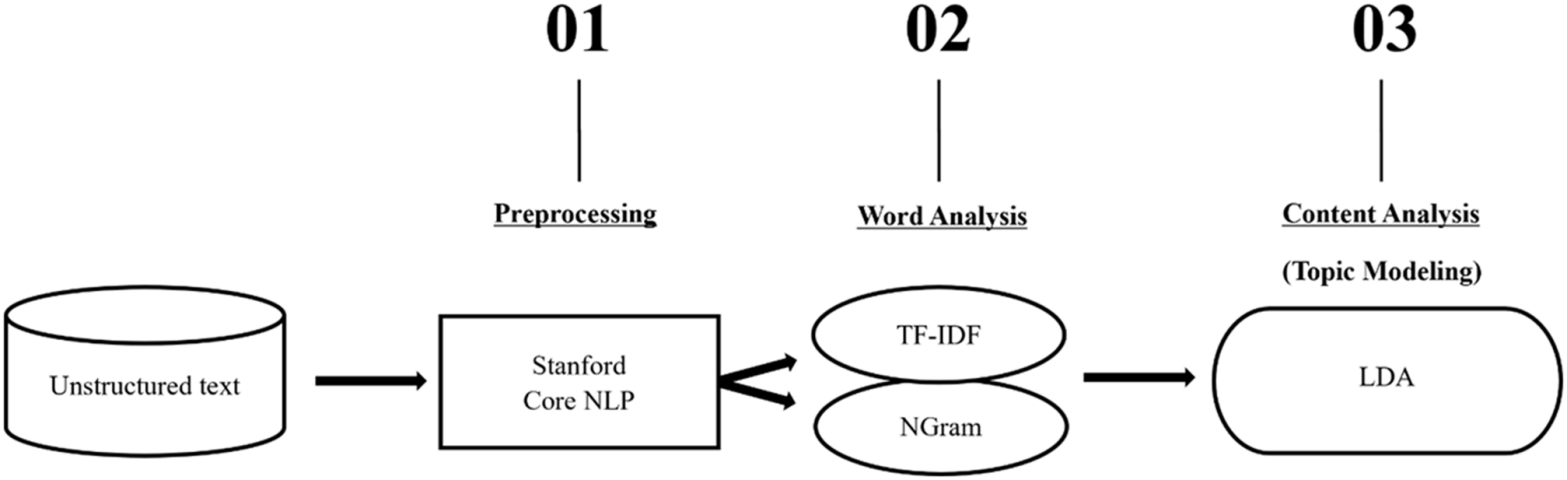
Text mining workflow. NLP: natural language processing; TF-IDF: Term Frequency-Inverse Document Frequency; N-gram: N-length sequence of words; LDA: Latent Dirichlet Allocation

For the primary text-mining analyses, we applied LDA, a key technique in topic modeling [21]to uncover hidden topics and patterns prevalent across documents related to active outdoor play. LDA assumes that each document is a mixture of multiple topics, and each topic is characterized by a distribution of words. Using this approach, we identified distinct topics by estimating the underlying structure of word co-occurrences, allowing us to capture latent themes within a collection of text data. The text-mining package utilized was yTextminer, a text mining pipeline written in Java [23], and the data were processed in Eclipse (Eclipse Foundation, n.d.). The specific text mining workflow is illustrated in Fig. 2. Of note, we conducted multiple preprocessing steps and used Term Frequency-Inverse Document Frequency (TF-IDF) outcomes to identify and filter any common or unrelated words (i.e., stop words). TF-IDF quantifies how important a word is to a document by assigning higher scores to words that appear frequently in a specific document but not in others [24]. We then performed multiple LDA analyses to gain clearer results through topic modeling. We set the model to extract 10 topics, with probabilities calculated based on (1) word-topic distribution (how often words associated with a given topic appear within a document) and (2) document-topic distribution (how often that topic appears across all documents in the dataset) [25]. 

#### Data collection for project scope – linked network analysis

To help visualize the inherent complexity among active outdoor play and cross-sectoral data in Canada, a linked network analysis was conducted (LV). Gephi (version 10.1; Bastian, M) was used for network visualization purposes, general analyses, and measuring network properties [26]. The essential data structure supported by Gephi are nodes, edges and attributes, where nodes and edges constitute the network topology, and attributes represent the network data.

##### Linked network analysis – data collection and processing

A database created and maintained by OPC was leveraged as a starting point for this network analysis as it contains a current and robust list of international partners and stakeholders in the physical activity/play and outdoor environment sectors. Members of the international leadership team (Supplemental File 1) were also consulted to help fill any apparent gaps in the dataset. Included in this revised dataset of extracted data were the names (i.e., target), locations, sector representation (i.e., type and attribution), strength of relationship and level of reciprocity for each organization contained in the list.

##### Linked network analysis – data analysis

All network analyses took place between July 2, 2023 and September 8, 2023, then again between July 6, 2024 and September 4, 2024, and October 8, 2024 and March 21, 2025. Once the database was imported, Gephi extracted every unique value or entity in the dataset to build a node list – i.e., an inventory of every node (or “circle”) that appeared on the network diagram. Every node was assigned a unique identification number, which by default was the same as the node name. A bimodal network graph was created, whereby a node was created for each organization (i.e., entity). Each node was connected by an edge—i.e., a visual indicator of how each of the nodes are related or connected. The weight of each relationship was defined by assigning a numerical value commensurate with the level of importance. Visually, the thickness of the edges indicated the weight of the connection, whereby the thickness of the line indicated the strength or value assigned to that particular relationship. Modularity (using the Louvain method) [27]—i.e., the measure of the strength of the network graph’s division into clusters—was calculated to identify groupings of sector organizations that had the most/least in common. A high modularity score indicates a network with well-defined communities, where nodes within a community are densely connected, while nodes in different communities have fewer connections. Conversely, low modularity suggests a weak community structure, where connections within and between communities are more evenly distributed. Modularity, a metric used to quantify community structure, ranges from − 1 to 1, with 1 representing a fully or high modular (well-defined communities) and − 1 representing a non- or low modular (random) network [27]. Once modularity was calculated, nodes were colored according to their clusters (i.e., communities), which permitted researchers to visualize which factors bound them together into communities. In other words, entities that had the most in common were assigned the same color (i.e., ‘alike’ organizations were assigned the same color based on sector attribution/representation).

### Conceptual framework and survey

Based on the above efforts, central themes and subthemes related to active outdoor play inclusive of all ages were compiled by LDL and OL and reviewed by the executive team, who developed a draft conceptual framework encapsulating these themes and subthemes. A Google Forms survey was then distributed to all steering committee members (*n* = 105 at the time) to assess level of agreement on the conceptual framework and provide an opportunity for additional feedback. The survey was open for three weeks in November-December 2023. Comments were compiled by LDL and E-YL and sorted according to the main themes of the framework. The executive team then each individually assessed all the feedback suggestions, scoring them using a color-coding system: green (include), yellow (maybe include), and red (reject). OL consolidated these individual scoring responses and re-sorted them into the following categories: all green (include), all red (reject), and all yellow comments as well as any mixed colors (e.g., green-green-yellow), were deemed to represent discordance among executive team members. Yellow or mixed color categories were reviewed by the executive team to collectively make a final decision on whether to keep or reject these suggestions. Suggestions were then integrated into the model before being sent to the full leadership group for final review and input.

### Overview of reviews used to inform the AOP10 project

Once the conceptual framework was developed, the executive team contacted all leadership team and steering committee members with an invitation to sign-up —via an online live spreadsheet—to lead and/or contribute to systematic reviews, scoping reviews, world region reviews and environmental scans falling under each of the conceptual framework themes. In the invitation, the executive team encouraged all potential authors to review the International Committee of Medical Journal Editors (ICMJE) criteria for authorship to ensure standardization of contribution, the details of which were included as a separate tab in the online spreadsheet.

Once all reviews were assigned a lead author and co-authors, the executive team contacted all lead authors with guidance on how to begin engaging their co-authors. Lead authors of systematic or scoping reviews were provided with a ‘Systematic Review Contributions Strategy’ file (Supplemental File 5) and encouraged to ask co-authors to identify at minimum one activity they would contribute to as part of the review. This file was developed by the executive team to assist with task assignment and workload distribution. In addition, the AOP10 leadership group, in collaboration with a librarian from the University of Ottawa (LS), developed an active outdoor play search strategy in May 2024, which was then peer-reviewed by a second librarian and reviewed by all leadership group members. This search strategy was circulated to all review authors to ensure the concept of active outdoor play was approached similarly across all systematic reviews being conducted. This search strategy was translated for use with MEDLINE, Embase, CINAHL, SportDiscus and SCOPUS. In August 2024, lead authors of systematic reviews were additionally provided with a data extraction template (Supplemental File 6) to ensure specific variables were being captured across all systematic reviews, to facilitate and streamline the data extraction process. Finally, lead authors were asked to provide a monthly update on the progress of their review to be shared at the monthly leadership group meetings and were additionally prompted to update an online review manuscript tracking sheet stored online via SharePoint.

The world region reviews were designed to focus on distinct geographic regions, organized according to the United Nations’ six geographic areas [28]: Africa, Asia, Europe, Latin America and the Caribbean, Northern America, and Oceania. Specific leadership team or steering committee members that were current residents of these areas were invited to contribute and/or lead these reviews. The lead author of the Northern America world region review (MST) circulated an outline to all other world region review authors to encourage similar structure and content across all narrative reviews and facilitate comparison across regions.

## Results

### Environmental scan

**Fig. 3:**
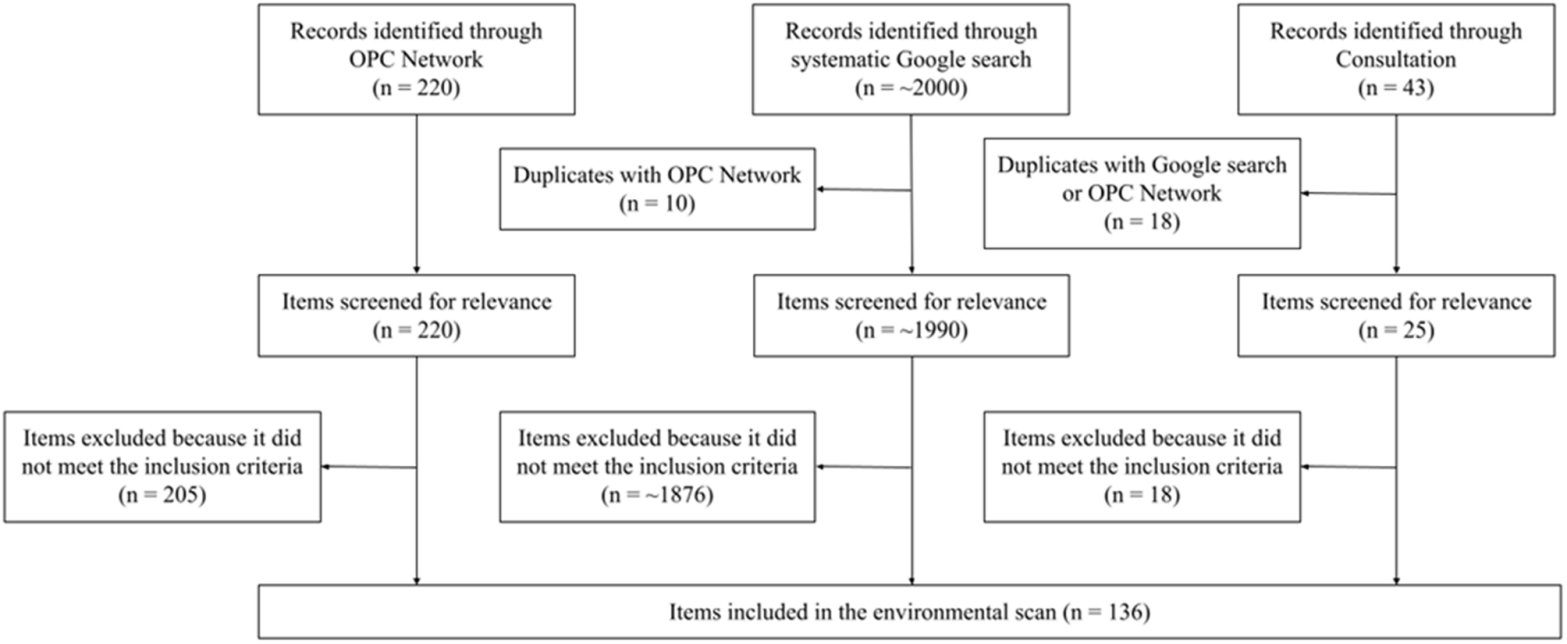
Flow diagram of items included in the environmental scan. *Note. Due to the nature of the Google search strategy, the exact number of resources that were screened could not be accurately determined. Thus, the number of resources indicated in Fig. 3 under ‘Records identified through the systematic Google search’ is an estimate based on the average number of links that were screened per the Google search

**Fig. 4:**
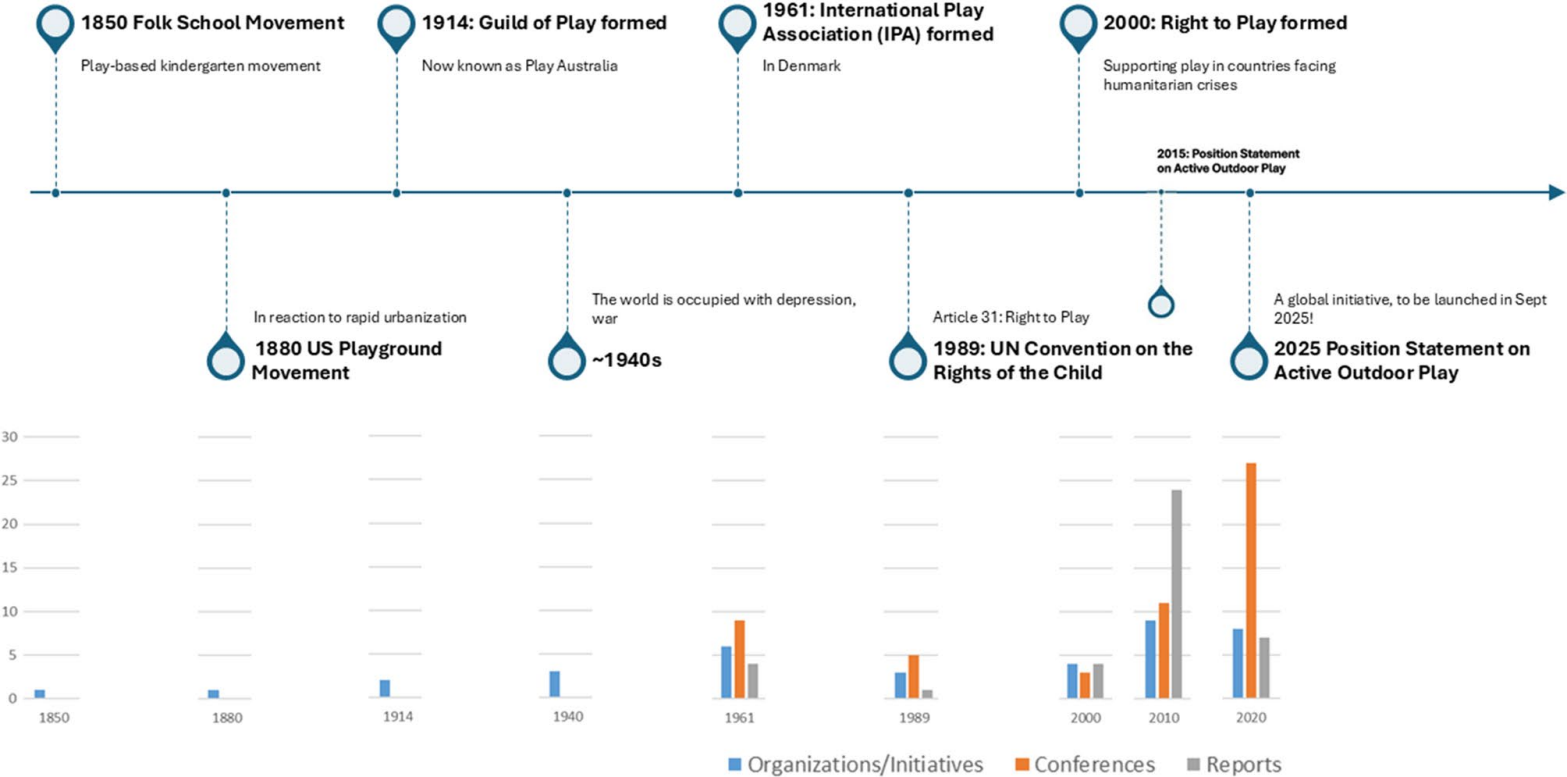
Visual depiction of the evolution of outdoor play. Major events are highlighted approximately every 30 years from 1850 to present day. Frequency of initiatives, conferences and reports that occurred and were produced within these 30-year time frames are shown as a bar graph directly below each highlighted event

The environmental scan included a total of 136 items that contributed to the development and/or progression of the global outdoor play sector, inclusive of all ages (Figs. 3 and 4). Items included ranged from the 19th century (i.e., 1801–1900) that started the outdoor play movement to present-day events (June 2023). Figure 4 provides a visual depiction of the evolution of outdoor play, highlighting major events and the frequency of initiatives, conferences and reports that occurred and were produced approximately every 30 years from 1850 to present day. Information on each item, including name, uniform resource locator or website link, and project summary was extracted and compiled in an Excel spreadsheet (Supplemental File 2). The full list of data, organized into a timeline, has been published online (https://www.sutori.com/en/story/environmental-scan-on-history-of-outdoor-play--GLEcuSQnQDpXWdUTakvhK6an). All included items were available in either English or French.

### ChatGPT

We submitted three prompts to Chat-GPT to identify common/main themes across previously published Position Statements related to active outdoor play [11, 12, 29–34] and EIOs from steering committee members (Supplemental Table 1). Supplemental File 3 shows the main themes and Table 1 indicates the overall summary provided by Chat-GPT.

**Table 1:** Chat GPT prompts and overall summaries provided

ChatGPT Prompt	ChatGPT Overall Summary
“What are the common/main themes among these Position Statements?”	Overall, these themes reflect a collective understanding of the importance of play in children’s lives and the need to create supportive environments and policies that enable all children to engage in outdoor play and learning experiences. The statements also underscore the role of collaboration and evidence-based practices in promoting positive outcomes for children’s development and well-being.
“What are the common/main themes among these Expressions of Interest?”	Overall, these common themes highlight the passion, expertise, commitment, collaboration, and potential impact of individuals who are interested in contributing to various projects and initiatives within their respective fields.
“What are the common/top areas of expertise/interest among these Expressions of Interest?”	Multiple individuals share expertise in promoting outdoor play and physical activity for children’s development and well-being. They possess skills in research methodologies, health promotion, and advocacy, often collaborating globally to advance equitable access, physical literacy, nature-based education, and child-centered approaches while translating impactful research across disciplines.

### Text mining

LDA topic modeling identified 10 distinct themes related to active outdoor play (Table 2). Each topic represents a set of co-occurring keywords that reflect academic discourse on active outdoor play, and these keywords were interpreted to generate meaningful topic labels. Rather than assigning fixed labels, our team compiled varied interpretations based on multiple perspectives. The topic probabilities were relatively similar (around 6–8%), indicating that academic discourse covers multiple themes evenly. However, among the identified topics, Cluster 1 (7.46%) emerged as the most prevalent, followed closely by Cluster 2 (6.99%) and Cluster 3 (6.72%). In contrast, Cluster 9 (3.61%) and Cluster 10 (3.49%) were less dominant, suggesting that while these topics are present, they are not as widely discussed in the dataset.

**Table 2:** Ten distinct themes related to active outdoor play identified through LDA topic modeling

Cluster	Topic Probability	Keywords	Compiled Interpretations
1	0.07461	child physical children play school parent intervention neighborhood active level sedentary childhood preschool environment family mvpa screen health social young adolescent sb playground behavior home parental adult youth obesity young_child preschooler gender risk change mother policy opportunity childcare healthy	a) Childhood Health and Active Play b) Healthy Childhood Development c) Youth Physical Activity d) Fostering engagement in outdoor physical activity and healthier lifestyle habits amongst preschool children and adolescents e) Encouraging physical activity and active outdoor play among children and adolescents
2	0.06994	nature student social role experience education natural teacher development play knowledge qualitative way practice environmental context review school learning game science place environment process understanding interview approach adventure concept systematic challenge interaction cognitive individual evidence benefit intervention theoretical systematic_review	a) Nature Education: A Systematic Adventure b) Educational and Developmental Benefits of Outdoor Play in Natural Environments c) Outdoor Education d) Self-learning and exploration through nature e) Educating students on the relationship between outdoor play and the environment
3	0.06722	urban nature environmental recreation public green human natural recreational green_space local park space role forest city resident ecological social landscape conservation species community spatial environment climate health management climate_change water rural ecosystem case respondent economic individual biodiversity large population	a) City Parks: Green Spaces for All b) Enhancing Urban Environments for Active Outdoor Recreation and Nature Interaction c) Community, Environment, and Nature d) Human health and well-being considerations of recreational use of public green spaces, in the age of climate change e) The impact of rural and urban community living on climate change
4	0.06141	physical covid older health older_adult covid_pandemic mental mental_health social injury risk intervention pandemic level participation patient change leisure home daily individual depression sport elderly factor wellbeing cognitive indoor psychological quality mobility active healthy lockdown type online gender anxiety community	a) Healthy Aging in the COVID Era b) Impact of the Pandemic on the Physical and Mental Wellbeing of Older Adults c) COVID-19, Injury, and Mental Illness d) The consequential effects of COVID-19 lockdown on the physical and mental health of older adults e) Determining the correlation of COVID-19 and health disparities, including mental health, among older adults
5	0.04577	myopia vitamin risk child prevalence sun risk_factor eye myopic school skin sun_exposure refractive refractive_error serum skin_cancer light level axial gender visual factor deficiency axial_length student uv myopia_progression ocular sun_protection questionnaire exposure status hour progression spherical_equivalent ohd odds_ratio parental summer	a) Child Myopia and Sun Exposure Risk b) Relationship Between Outdoor Activity, Sun Exposure, and Eye Health in Children c) Visual Disorders d) The health benefits and consequences of sunlight exposure: vision health, vitamin D, and skin cancer e) The risk of developing myopia and skin cancer among children with increased sun exposure
6	0.04062	indoor air pm exposure air_pollution concentration air_quality personal mu particulate_matter health pm_concentration indoor_air ambient home human particulate urban particle source level personal_exposure mu_gm air_pollutant soil daily asthma risk indoor_pm population traffic pollutant model health_risk respiratory environmental ventilation city period	a) Indoor Air Quality and Health b) Understanding the Health Implications of Outdoor Play in Urban Environments with a Focus on Air Quality and Particulate Matter Exposure c) Air Pollution d) The health risks of poor air quality in urban environments e) The impact of poor air quality and high levels of pollution among urban populations
7	0.03946	thermal temperature thermal_comfort heat degree energy air summer winter indoor performance climate hot solar building weather cold human air_temperature space thermal_environment condition wind physiological water model comfort thermal_sensation field relative_humidity extreme experimental urban seasonal meteorological change environmental heat_stress solar_radiation	a) Climate Comfort b) Optimizing Thermal Comfort for Safe and Enjoyable Outdoor Play c) Climate and Weather d) Optimal indoor thermal temperature during different seasons e) Promoting overall performance through determining an optimal thermal temperature based on seasonal conditions
8	0.03639	role nature plant protein surface temperature species growth development interaction complex mechanism biological property water material soil app host presence energy light process structural structure type concentration function degree experimental natural ph large expression field active diverse review size	a) Biological Role of Surface Plant Proteins b) Complex Interactions Between Natural Environments and Child Development c) Botany d) Biological terms associated with plant growth e) Determining appropriate biological mechanisms to explain the interaction and associated outcomes of plant processes
9	0.03613	patient risk case tick human risk_factor clinical infection disease animal dog exposure role incidence access diagnosis common cat individual indoor population prevalence behavior blood presence symptom endemic allergic wild lyme adult pig transmission infectious factor environmental tbe tickborne prevention	a) Tick-Borne Diseases b) Assessing Health Risks Associated with Outdoor Play in Tick-Prone Environments c) Infection and Disease d) The environmental risk factors of tickborne viruses and their impact on human health e) The associated risk of environmental tickborne exposure and perceived health outcomes among patients
10	0.03487	system malaria model indoor human mosquito user performance app approach image environment accuracy prediction mobile real neural_network wearable development device sensor feature accurate framework complex role information machine deep classification task smart spatial detection field effective type monitoring anopheles	a) Smart Malaria Detection System b) Innovative Technology and Data-Driven Solutions for Malaria Prevention during Outdoor Activities c) Modeling Technology d) Development of wearable technology and app model to enhance user performance e) Utilizing wearable technology to determine the accuracy of user performance outcomes through spatial detection and sensor features

**Table 3:** List of Systematic, Scoping and World Region Reviews Included in the AOP10 Project*

Review Title	Conceptual Framework Theme(s)	Protocol Registration/Publication
**12 Reviews**		
Systematic review of the association between outdoor play and the 24-hour movement behaviours among children, youth and adults	Movement Behaviors	https://www.crd.york.ac.uk/PROSPERO/view/CRD42024517145
Associations between active outdoor play and health and wellbeing among children, adolescents, and adults: an umbrella review	Health and Well-being	https://www.crd.york.ac.uk/PROSPERO/view/CRD42024565295
What is the relationship between outdoor risky play and health in children? Results from a systematic review	Health and Well-being	https://www.crd.york.ac.uk/PROSPERO/view/CRD42023488023
A mixed-methods systematic review of the association between active outdoor play and environmental stewardship outcomes among children, youth, and adults	Nature and Environment	https://www.crd.york.ac.uk/PROSPERO/view/CRD42024552064
Climate change and active outdoor play: a systematic review and qualitative synthesis	Nature and Environment	https://www.crd.york.ac.uk/PROSPERO/view/CRD42024560103
The PLAY+ (Play, Land, Animals, You and +) Framework: the role of active outdoor play in advancing One Health	One Health / Community, Connection, & Partnerships	Not available
Active play among young children (0–4 years) with disabilities: a scoping review	Human Rights and Policy / Community, Connection, & Partnerships	10.17605/OSF.IO/GWPSD
Places and spaces for play among children and youth with disabilities: an umbrella review	Human Rights and Policy / Community, Connection, & Partnerships	Not available
‘The state of play in outdoor play’ - Exploring global Indigenous knowledge of outdoor play: a scoping review	Human Rights and Policy / Community, Connection, & Partnerships	Not available
Systematic review and qualitative meta-synthesis on (active) outdoor play and social capital: relationships and impacts	Social Capital / Community, Connection, & Partnerships	https://www.crd.york.ac.uk/PROSPERO/view/CRD42024568624
Teacher implementation of active outdoor play-based learning: a systematic review of pedagogical models and practices	Education and Learning	https://www.crd.york.ac.uk/PROSPERO/view/CRD42024551581
An environmental scan of global outdoor play-based projects, programs, and initiatives	Community, Connection, & Partnerships	Not available
**World Region Reviews**		
Outdoor play in the Africa: a status update	Human Rights and Policy / Community, Connection, & Partnerships	Not available
Outdoor play in the Asia region: a status update	Human Rights and Policy / Community, Connection, & Partnerships	Not available
Outdoor play in Europe: terminology and state of research, practice, and policy	Human Rights and Policy / Community, Connection, & Partnerships	Not available
Outdoor play in the Latin America and Caribbean region: a narrative review on the challenges, lessons learned, and recommendations for the future	Human Rights and Policy / Community, Connection, & Partnerships	Not available
Outdoor play in the Northern America region: a status update	Human Rights and Policy / Community, Connection, & Partnerships	Outdoor Play Canada
Outdoor play in Oceania: a review of policy, advocacy, and regional priorities	Human Rights and Policy / Community, Connection, & Partnerships	Not available

*Note: These reviews are currently underway, and the title may be subject to change during the review process

### Linked network analysis

#### Initial network Analysis – Month 0 (Fig. 5A)

**Fig. 5:**
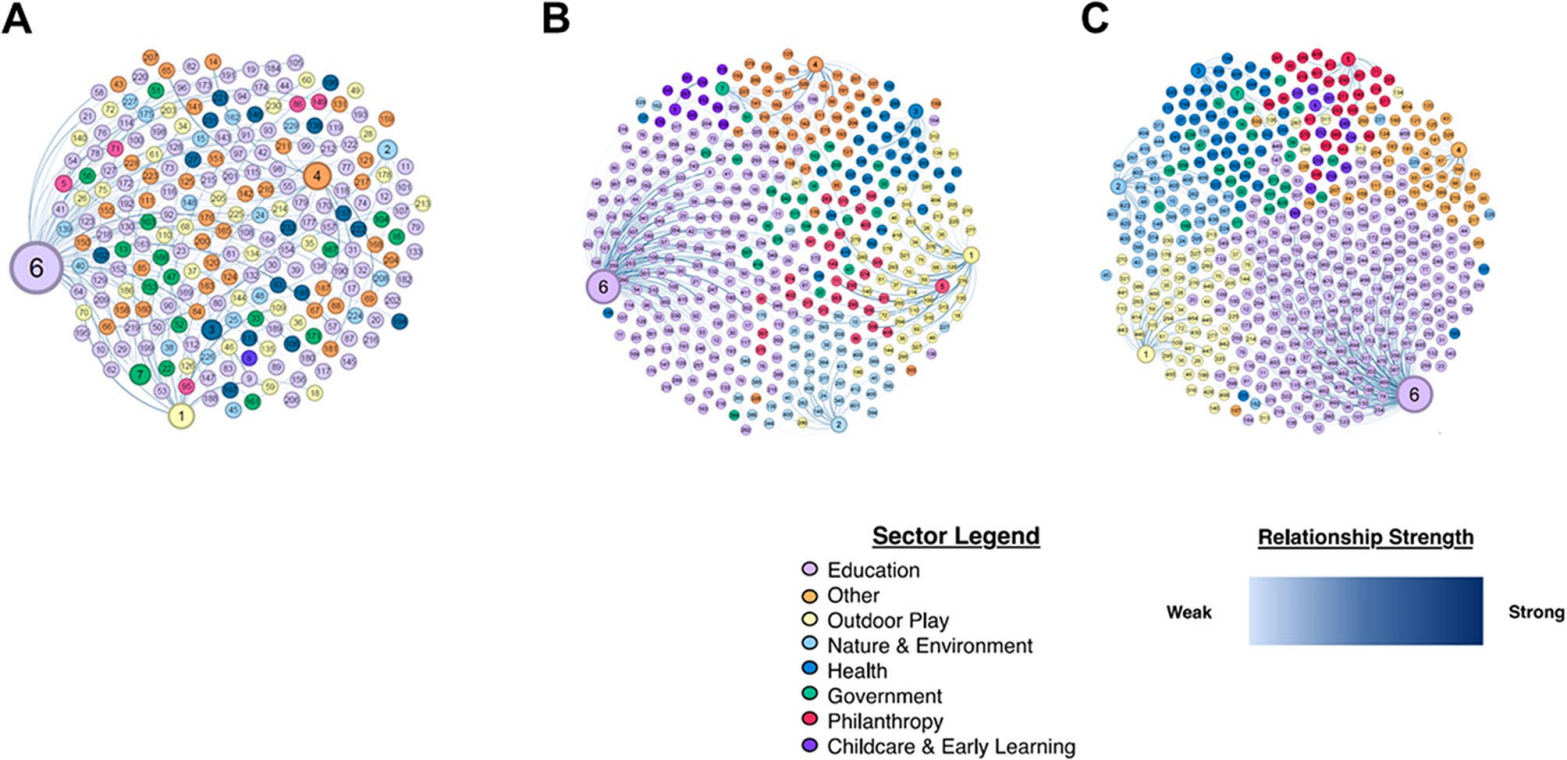
Linked Network Analysis of Organizations and Individuals in the field of Outdoor Play in Canada, by Sector. **A**: Analysis conducted from July 2023 to September 2023. **B**: Analysis conducted from July 2024 to September 2024. **C**: Analysis conducted from October 2024 to March 2025

The results of the linked network analysis highlight the strength of relationships among numerous organizations related to active outdoor play. The nodes represent the entities and are classified by sector, which form clear clusters. These clusters include physical activity, nature and environment, health, philanthropy, education, childcare and early years environments, government and other. The edges (i.e., lines between nodes) represent the relationship’s strength based on their weight determined by contact frequency within the last 12 months. The weights of the connections were assigned a number from one to five and range from very strong, strong, moderate, weak, to very weak. New contacts and non-existent connections were also included but were not assigned a weight. The average weighted degree for this network map was 4.2. The thicker and darker edges suggest a greater weight, which indicates a more robust and symmetrical relationship. Isolated nodes can be seen throughout the network map, contributing to its low density of 0.007. These nodes were classified as either new contacts or non-existent connections. This demonstrates an opportunity to re-evaluate the necessity of those connections, rethink partnerships and create new ones if needed. The network analysis’ modularity was deemed moderate, with a score of 0.742, insofar as a dense connection to the nodes in the same cluster with few connections to other groups is evident.

The network also demonstrates that, at first glance, the education sector is currently most likely a key player in active outdoor play, representing 47.2% of all nodes. While it has the most significant number of entities, 40.0% of these entities can be categorized as weak, very weak, or non-existent relationships. Sectors with fewer entities, such as health, had 50.0% of their relationships classified as very strong or strong with no isolated nodes. The health, philanthropy, and governmental sectors comprise only 14.0% of the total number of nodes, which might indicate a possible underutilization in active outdoor play.

#### Second network analysis – month 12 (Fig. 5B)

Following the initial linked network analysis created in September 2023, the list of contacts in the outdoor play sector in Canada grew substantially. Therefore, a second linked network analysis was performed in September 2024 to identify the key similarities and differences at the twelve-month mark. Once again, the network’s density remained low at 0.004, indicating a lack of interconnectedness between nodes. The modularity remained similar, with a score of 0.739, alluding to more robust clusters throughout the visual. The average weighted degree remained unchanged at 4.2.

While the nodes still represent the primary sectors of the key players in the active outdoor play space, a new sector called childcare and early learning was included in the updated version. There was now a total of 417 nodes compared to the 232 nodes in the initial analysis. The primary sector continued to be education, with 180 (43.2%) nodes. The sectors with the most significant growth included philanthropy, outdoor play, and nature and environment. While they initially comprised 22.0% of all nodes, they now made up 30.0% of all nodes. The sector with the smallest number of nodes was childcare and early learning, with only 2.6% of all nodes. This could demonstrate an underutilization of key players in this sector.

Though childcare had the fewest number of nodes, it had the most significant percentage of relationships categorized as strong or very strong, at 27.2%, followed by outdoor play at 26.4%. This was a shift from the initial analysis, where health had the most significant percentage of relationships categorized as strong or very strong, at 50.0%. The increased number of entities included in the linked network analysis allowed for a more robust and complete picture of the organizations involved in active outdoor play across Canada. However, 59.4% of all relationships across sectors were categorized as weak or very weak. A focus on strengthening relationships across different sectors could lead to a higher density, increased interconnectedness and shared expertise.

#### Third network analysis – month 18 (Fig. 5C)

The third and final linked network analysis was performed in March 2025 to identify the emerging similarities and differences at the 18-month mark. As with the 12-month update, the network’s density remained low at 0.004, reiterating the lack of connectedness between the nodes. The modularity also remained similar at 0.715 but had decreased slightly with the increase in the volume of nodes. However, the average weighted degree had increased to 4.9 compared to 4.2 in the first two initial network analyses, indicating the stronger relationships throughout the network.

In March 2025 there were 486 nodes in total, an increase of 254 from the first initial analysis and 69 from the 12-month update. The primary sector was once again education, with 225 (46.3%) nodes, an increase of 119 nodes from the initial analysis. The sector with the smallest number of nodes continued to be childcare and early learning, with only 12 (2.5%) nodes compared to none in the first analysis. Increasing the utilization of key players in this sector could potentially positively impact the area of active outdoor play among children.

The sectors with the most significant percentage of relationships categorized as strong or very strong were nature, at 51%, and outdoor play, at 27.6%, which differs from the 12-month update, when childcare and early learning held this distinction. A total of 49.8% of all relationships across sectors were categorized as weak or very weak, indicating an almost 10% improvement compared to the 12-month analysis completed in September 2023. In March 2025 the education sector contained the largest number of nodes and had the largest number of relationships categorized as weak or very weak, at 53%, highlighting a chance to re-evaluate and strengthen partnerships. While the number of key players increased exponentially since the first analysis, along with an increase in the average overall strength of relationships, a focus on continuing to fortify current, and add new, partnerships would be beneficial long-term in the active outdoor play space in Canada.

### Conceptual framework

**Fig. 6:**
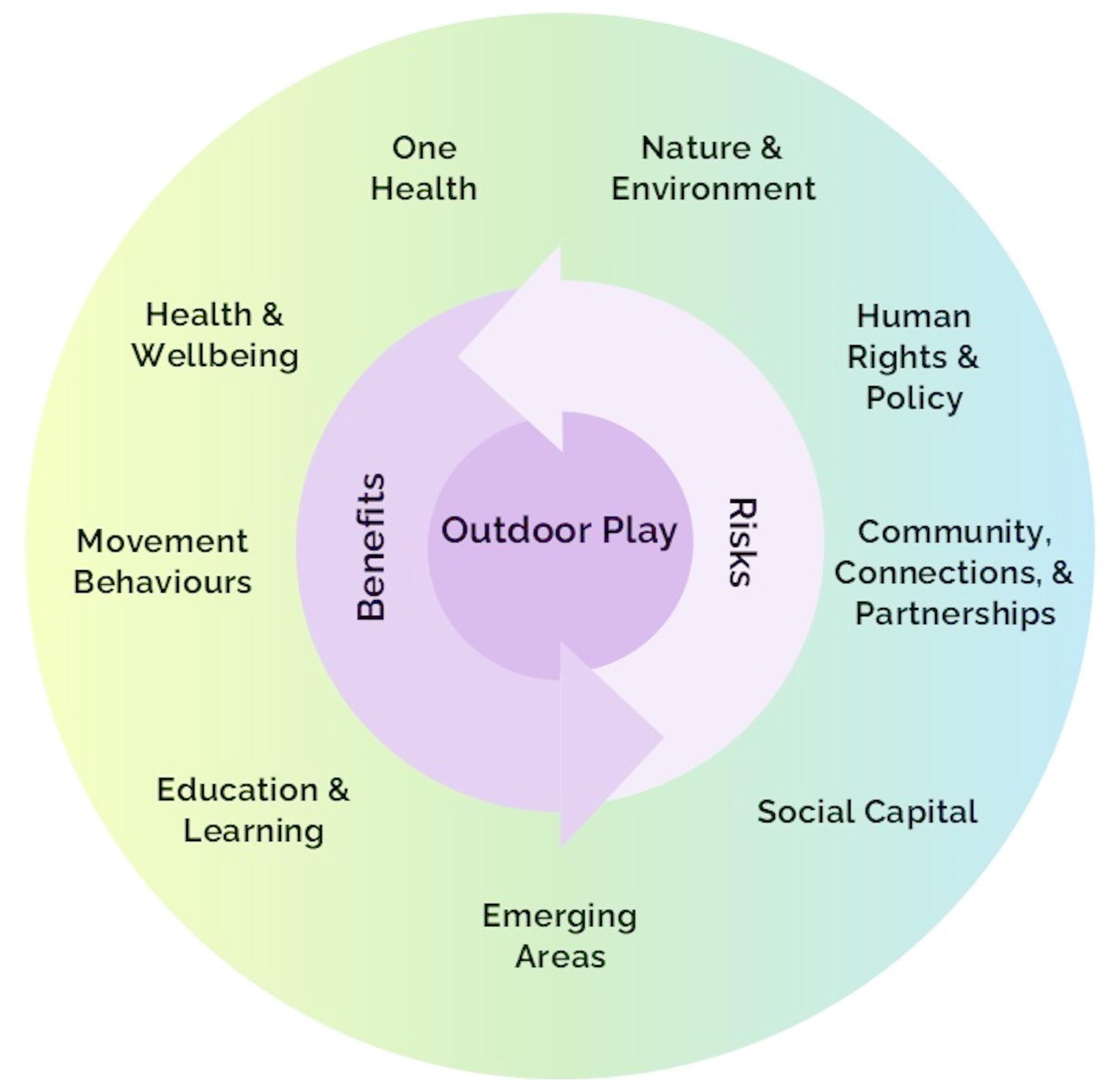
Conceptual framework for the 10-year anniversary update of the Position Statement on Outdoor Play Project. The outer green-blue circle contains central themes related to outdoor play, where all themes have overlapping relationships. In the middle, surrounding the purple ‘Outdoor Play’ circle, are light and dark purple concentric overlapping arrows indicating benefits and risks, to indicate that all themes have benefits and risks associated with outdoor play, where risks can beget benefits and vice versa. Further description of the themes are as follows: *Education & Learning*: Several position statements on outdoor play and learning emphasize the positive impact of outdoor play and learning on academic performance, education outcomes, and high-quality educational experiences. Subthemes include cognitive development, outdoor learning spaces, curriculum assessment, student autonomy, Land-based play, reconciliation, reciprocal relationship between learning and outdoor play, learning through play/play pedagogies, and education across the lifespan. *Movement Behaviours*: The 2015 Position Statement on Active Outdoor Play highlighted that when children are outdoors, they move more, sit less and play longer. The 24 h movement behaviours include moderate-to-vigorous physical activity, light physical activity, sedentary behaviour, and sleep. Additional subthemes include physical literacy, active transportation, and competing interests between indoor activities (e.g., screen time, technology) and outdoor play. *Health & Wellbeing*: The importance of outdoor play for physical, mental, social, emotional health and wellbeing are well-established. However, these benefits are becoming ever more complicated by existing and emerging environmental risks (e.g., poor air quality, particulate matter exposure, extreme weather, sun exposure, diseases). Subthemes include: eco-anxiety, mindfulness, injury, growth and development. *One Health*: One Health is an interdisciplinary approach to combatting global health issues related to human and animal health, as well as biodiversity, ecology, climate change, agricultural systems and various social sciences. It recognizes that humans, plants and animals share the same planet, the same environmental challenges, and overlapping challenges to health. *Nature & Environment*: Outdoor play is associated with (and can lead to the development of) a connection to nature and place. Subthemes related to this theme include environmental awareness, eco-anxiety, place, Land, nature literacy, sustainability, planetary health, stewardship, balance between preserving access to spaces and conservation, design of purpose-built outdoor spaces, and accessibility of public outdoor spaces. *Human Rights & Policy*: Article 31 of the United Nations Convention on the Rights of the Child recognizes play as a fundamental right of the child, which needs to be recognized in policy development and urban planning. This theme recognizes the importance of Article 31, while also recognizing that outdoor play is not limited to children. Subthemes include equity and inclusion, Indigenous perspectives, reconciliation, justice, social justice, spatial justice, sustainable development goals, self-awareness, agency, inequalities, freedom, accessibility, opportunity (time and access), peace, rights of persons with disabilities, climate action, child agency/autonomy, and policy (legislation, guidelines, strategies, licensing/regulation). *Community*,* Connections*,* & Partnerships*: Promoting, protecting and preserving access to play in nature and the outdoors for all requires collaboration between policymakers, researchers, organizations, educators, stakeholders, Indigenous partners, children, youth, and the community. Subthemes include advocacy and leadership, community building/placemaking/heritage, culture, inclusion, policy, access to play, the role of government institutions and agencies, and parents/partners/caretakers/guardians as key actors. *Social Capital*: Outdoor play has positive impacts on social development and shaping social relationships (e.g., making friends, learning rules, conflict resolution). Subthemes include family connections/bonding, social cohesion, safety, risk taking, resilience, critical thinking, problem solving, decision making, and cultural sustainability. *Emerging Areas*: Our intention is for this to be a living framework. We know we do not know everything that is related to outdoor play and welcome its evolution

An initial draft of the AOP10 conceptual framework based on themes identified through the above processes (i.e., environmental scan, LLM analysis, text mining analysis, linked network analysis) was circulated via an online survey to the steering committee, where respondents were asked the question: *‘Do you like the overall design of the framework?’.* The conceptual framework received 90% agreement, and a total of 87 comments were received (Supplemental File 7). Figure 6 depicts the final version of the AOP10 conceptual framework.

### Systematic, scoping, and narrative reviews

The conceptual framework was then used to guide the focus of background systematic, scoping, and narrative reviews, to be used to inform the final 2025 Position Statement on Active Outdoor Play. The final list of reviews is provided in Table 3.

## Discussion

The combined outputs of the leadership group’s discussions, environmental scan, text mining analysis, linked network analysis, conceptual framework, and the foundational reviews form a well-informed basis for the 2025 Position Statement on Active Outdoor Play. Through this process, the leadership group identified nine major themes that have bidirectional relationships with active outdoor play, which led to the launch of 18 reviews exploring these themes, all serving to inform the 2025 Position Statement. This background work aimed to ensure a globally relevant and evidence-based update while facilitating international collaboration and engagement, to position the AOP10 project as a landmark initiative for promoting active outdoor play globally and for all ages.

Through this process, the leadership group identified several avenues to extend and update the 2015 Position Statement. This project aimed to examine the benefit of active outdoor play across all ages (bringing outdoor play into the vernacular for adult populations) and make the update global in reach by recognizing and incorporating diverse cultural, environmental, and policy contexts surrounding active outdoor play, including global considerations related to access and equity. Moreover, while the 2015 Position Statement focused largely on the value of outdoor risky play for children’s health, development, and resilience, the 2025 update recognizes the need to shift focus towards addressing active outdoor play within the context of multiple, major global challenges including climate change, urbanization, physical inactivity, digital addiction and epidemics/pandemics. The need for this shift was recognized in part through the text mining analysis, which highlighted that environmental risks, including pollution and disease exposure, are widely discussed in the field of active outdoor play. Moreover, this analysis highlighted potential disparities in accessing the benefits of outdoor play based on residential, geographic, sociodemographic, ethnic, racial and/or ecological factors, and that certain populations face greater barriers due to environmental exposure, socioeconomic factors, health vulnerabilities, and urban-rural divides. Collectively, these findings indicate the need for adaptable outdoor play policies and interventions that not just account for climate-related, ecological, and health risks but serve to address these inequities, to help promote safe and equitable access to outdoor spaces for play and recreation across all people at all ages.

The decision to focus on all ages is in alignment with the mission and vision of OPC that outdoor play be a valued part of daily life for all people. It is also in alignment with the definition of active outdoor play from the PLaTO-Net project: *Voluntary engagement in activity that is fun and/or rewarding and usually driven by intrinsic motivation*,* that takes place outdoors and involves physical activity of any intensity* [15]. However, the notion that active outdoor play initiatives and publications remain largely focused on children was emphasized in the LLM analysis where the summary provided on previous Position Statements and areas of interest of steering committee members both focused solely on active outdoor play in relation to children and childhood. How to discuss active outdoor play beyond childhood was a frequent topic of discussion by systematic review co-authors contributing to this project. For example, many conversations have been —and continue to be —held on whether sport can be considered play. For children, it is difficult to determine whether organized sport is self-motivated and was thereby excluded from our definition. However unorganized, spontaneous games of child-led soccer or baseball is often the main way that children play and therefore should be considered play. Recreational outdoor sports clubs for adults would also arguably be considered play, as these are often self-refereed and played for few reasons other than enjoyment and social engagement. The executive team for this project recognized that though active outdoor play is not something commonly associated with adulthood, evidence of its benefit across all ages [35, 36] informed the decision to make this Position Statement inclusive of all ages. This decision was supported by the results of the text mining analysis, which showed that while the role of outdoor play in childhood health and wellbeing is well-established, emerging evidence of the benefits of outdoor play for adults and older populations suggests the need to expand research and policy discussions beyond childhood. We recognize that such a notion stretches conventional boundaries and thinking – but such is the case when changes challenge the status quo.

In line with the spirit of the 2015 Position Statement, this update serves to bring together the many diverse groups involved, interested, and invested in efforts related to active outdoor play, including those from research, practice, policy, public health, medicine, advocacy, education, philanthropy, and community partnerships, among others. The results of the LLM analysis highlighted the already intersectoral nature of our steering committee, suggesting that our early efforts to bring these diverse groups together was successful. Similarly, the linked network analysis conducted as part of this process highlighted the multiple sectors involved in this work in Canada, and the continued expansion of this network, though it also emphasized the ongoing challenges of connecting these groups, as indicated by a low network density and thus a lack of interconnectedness between sectors across all three times points. The LNA served to highlight the growing impact of the 2015 Position Statement [29] in bringing diverse groups together in Canada, leading to an explosion of research evidence [6, 7], a proliferation of philanthropic investment [35], and the creation of new position statements [11, 12] by groups not represented in the initial Position Statement, thus supporting the rationale for this global update. It is our aim that through this internationally collaborative process, active outdoor play will become better recognized as having a role in bridging sectors and promoting collective action at a global scale.

### Strengths and limitations

A major strength of this project was its international representation across the leadership group and steering committee to meet our mandate of making this project global in scope. This meant that we had substantive international and intersectoral peer review of our entire process, from forming our leadership and steering committee groups, to creating the conceptual framework and developing an evidence-gathering plan. This helped ensure the robustness of our work. Finally, this project was informed by the 2015 Position Statement process, which was highly successful in galvanizing the outdoor play sector in Canada – such is our hope with this 2025 Position Statement at a global scale.

A limitation of this project is that, despite considerable effort not to limit our evidence-gathering to English-only efforts, English-speaking projects, updates, initiatives, and publications were dominant throughout this process. This was evident with our environmental scan, in which, despite an effort to conduct an online search of outdoor play initiatives using a private web browser and turning off location trackers, most search results were in English. Moreover, the linked network analysis was focused on Canada – the intention was to identify the impact of the 2015 Position Statement and related efforts on the expansion of the Canadian outdoor play network, providing rationale for the launch of this update. Nonetheless this analysis adds to the Canada-centredness of this work. The AOP10 leadership team is in the process of conducting a global linked network analysis to identify the expansion of the global outdoor play network since the start of the AOP10 project in 2023. This global analysis is being prepared as a separate manuscript and will serve to highlight the scope of global involvement in this work. Finally, our team is not free from bias in terms of the direction and way that this project was led, given that it was led by a predominantly pro-outdoor play Global North collective. Our processes undoubtedly followed a Western approach, reinforced by our often-tight timelines. Nonetheless, considerable effort was made to include voices beyond the Global North, by for example, our snowball invitations to join the steering committee via existing contacts in the Global South. We will be translating the Position Statement global consensus survey and all related final products into all six United Nations official languages to broaden the reach and representation of this work. Further, the Indigenous play systematic review was launched in an effort to include Indigenous perspectives, knowledge systems, and ways of being in this work.

### Future directions

This manuscript outlines the process involved in developing the resources and manuscripts used to inform the 2025 Position Statement on Active Outdoor Play. The process of developing a draft Position Statement and building and achieving international consensus on the final Position Statement is published as a separate manuscript^37^. The intention of this manuscript was to provide a transparent overview of our robust process, for the purpose of historical documentation and so that it may be used by others seeking to pursue similar efforts bringing together intersectoral and international colleagues to address many of the complex global challenges we all face.

Though the resources and manuscripts described here serve to inform the updated Position Statement, the intention is for these products to have far reaching implications beyond this project. We aim to create a robust list of recommendations, gaps in literature and action items, identified through the 18 reviews conducted and used to inform this project. Through this robust list, we hope to inspire connection, collaboration, and innovation within the outdoor play sector and with new sectors that through this process may now see themselves aligned with outdoor play. This is in alignment with the nineth theme of the conceptual framework guiding this project, ‘Emerging Areas’, which was added in recognition that not all connections to active outdoor play have been identified within this framework. From the start of this project, it has always been our hope and expectation that this global effort will shed light on ongoing gaps and new areas for future research, connection, and collaboration.

## Conclusions

The aim of this project was to outline the process of developing the 2025 Position Statement on Active Outdoor Play and have it serve as a guide for others aiming to pursue similar efforts in other fields. Twelve systematic reviews and six narrative reviews were led by our international leadership group and steering committee that informed the development of the initial 2025 Position Statement on Active Outdoor Play draft. Through this robust, international and dynamic process, we hope to set-up the 2025 Position Statement on Active Outdoor Play as an inspiring, innovative, future-looking document.

## Electronic supplementary material

Below is the link to the electronic supplementary material.

**Supplementary Material 1**: **Supplemental file 1**: Title: AOP10 Leadership Team and Steering Committee Members. Description: Full list of AOP10 leadership team and steering committee member names, affiliated countries and organizations. **Supplemental file 2**: Environmental Scan to Inform the AOP10 Project Scope. Description: Summary of environmental scan results and table describing the scan results based on item type (e.g., an outdoor play movement, organization, conference, event, project or document). **Supplemental file 3**: Summary Statements Developed by ChatGPT to Inform the AOP10 Conceptual Framework. Description: Table outlining the common/main themes of identified position statement and expressions of interest, summarized by ChatGPT. **Supplemental file 4**: Text Mining Search Strategy. Description: Table outlining the search strategy used for the text mining analysis identifying common themes related to active outdoor play. **Supplemental file 5**: Systematic Review Contributions Strategy. Description: Table outlining the strategy to ensure equitable contributions to systematic reviews among large author groups as part of the AOP10 project.

**Supplementary Material 2**: **Supplemental file 6**: Systematic Review Data Extraction Template. Description: Template outlining required and recommended data to extract per systematic review being led as part of the AOP10 project.

**Supplementary Material 3**: **Supplemental file 7**: Conceptual Framework Feedback from the AOP10 Steering Committee. Description: File listing feedback received from the AOP10 steering committee on the AOP10 conceptual framework.

## Data Availability

Data is provided within the manuscript or supplemental information files.

## Abbreviations

AOP10: Active outdoor play 10–year anniversary
APC: Outdoor play canada
GPT: Generative pre-trained transformer
CHEO: Children’s hospital of eastern Ontario
EOI: Expression of interest
LDA: Latent dirichlet allocation
LLM: Large language model
NLP: Natural language processing
PLaTO-Net: Play, learn, and teach outdoors network
SDGs: Sustainable development goals
TF-IDF: Term frequency-inverse document frequency
