# Supplementary material for: 2025 position statement on active outdoor play: process and methodology
Source: Int J Behav Nutr Phys Act. 2025 Sep 25;22:118. doi: 10.1186/s12966-025-01806-8 (PMC12462215; doi:10.1186/s12966-025-01806-8)
Supplement: Supplementary file 1 — Supplementary Material 1: Supplemental file 1: Title: AOP10 Leadership Team and Steering Committee Members. Description: Full list of AOP10 leadership team and steering committee member names, affiliated countries and organizations. Supplemental file 2: Environmental Scan to Inform the AOP10 Project Scope. Description: Summary of environmental scan results and table describing the scan results based on item type (e.g., an outdoor play movement, organization, conference, event, project or document). Supplemental file 3: Summary Statements Developed by ChatGPT to Inform the AOP10 Conceptual Framework. Description: Table outlining the common/main themes of identified position statement and expressions of interest, summarized by ChatGPT. Supplemental file 4: Text Mining Search Strategy. Description: Table outlining the search strategy used for the text mining analysis identifying common themes related to active outdoor play. Supplemental file 5: Systematic Review Contributions Strategy. Description: Table outlining the strategy to ensure equitable contributions to systematic reviews among large author groups as part of the AOP10 project. [file 12966_2025_1806_MOESM1_ESM.docx]

**SUPPLEMENTAL FILES 1-5**

**SUPPLEMENTAL FILE 1 – AOP10 Leadership Team and Steering Committee Members**

| **Name** | **Home Country** | **Organizational Affiliation** |
| --- | --- | --- |
| **AOP10 Leadership Team Members** | | |
| Dina Adjei Boadi | Ghana | University of Ghana |
| Isabel de Barros | Brazil | Alana Institute |
| Louise de Lannoy | Canada | Outdoor Play Canada |
| Scott Duncan | New Zealand | Auckland University of Technology |
| Maeghan James | Canada | CHEO Research Institute |
| Eun-Young Lee | Canada, South Korea | Queen's University, Play, Learn, and Teach Outdoors-Network |
| Robyn Munro Miller | Australia | International Play Association |
| Lærke Mygind | Denmark | Center for Clinical Research and Prevention, Bispebjerg and Frederiksberg Hospital |
| Mark Tremblay | Canada | CHEO Research Institute, Outdoor Play Canada, Play, Learn, and Teach Outdoors-Network |
| Leigh Vanderloo | Canada | ParticipACTION |
| Po-Yu Wang | Taiwan | National Taiwan Normal University |
| **AOP10 Steering Committee Members** | | |
| Alethea Jerebine | Australia | Deakin University |
| Alex Treolar | Australia | Monash University |
| Alexis Zeus | Canada | Compass |
| Alison Amero | Canada | Sport3 |
| Alyson Beben | Canada | Active for Life |
| Amanda O'Rourke | Canada | 880 Cities |
| Amy Latimer-Cheung | Canada | Queen's University |
| Anna Haanstra | Canada | Government of Nova Scotia |
| Anujah Thankarajah | Canada | University of Guelph |
| Areekul Puangsuwan | Thailand | University of Western Australia |
| Arlene Lusterio | Canada | Embrace Health |
| Arlene McGarty | Scotland | University of Glasgow |
| Ashley McCurdy | Canada | University of Alberta |
| Avril Johnstone | Scotland | University of Glasgow |
| Benjamin Billet | France | European Network of Outdoor Sports |
| Carla Gull | USA | Merry Lea Environmental Learning Center |
| Chris Speldewinde | Australia | University of Melbourne |
| Christine Alden | Canada | Lawson Foundation |
| Colin Harris | Canada | Take Me Outside |
| Colleen Kiselyk | Canada | Pembina Hills School |
| Damien Puddle | New Zealand | Hamilton City Council |
| Dané Coetzee | South Africa | North-West University |
| Daniel Burton | Australia | Educated by Nature |
| Danielle Wittick | Canada | NYAD Community Inc |
| David Chorney | Canada | University of Alberta |
| David McFall | Canada | PETES school |
| Diego Augusto | Brazil | Federal University of Santa Catarina |
| Ellen Sandseter | Norway | Queen Maud University |
| Emily Payne | Canada | Rivers to Ridges |
| Emily Recoskie | Canada | Compass |
| Erica Phipps | Canada | Canadian Partnership for Children's Health and Environment (CPCHE) |
| Erin Wentzell | USA | Hopkins Hospital and George Washington University |
| Ernesto Martire | Italy | Francesco Tonucci |
| Franz Plangger | Canada | Outdoor Council of Canada |
| Frederick Marais | South Africa | Western Cape Department of Health |
| Frederico Lopes | Portugal | University of Lisbon |
| Gemma Goldberg | UK |  |
| George Owusu | Ghana | University of Ghana |
| Gill Pomfret | UK | Sheffield Hallam University |
| Heather McGregor | Canada | Queen's University |
| Helen Dodd | UK | Reading University |
| Hyunshik Kim | Japan | Sendai University |
| Inspiring Scotland's Thrive Outside Team | Scotland | Inspiring Scotland |
| Jade Berrill | Canada | Outdoor Learning Store |
| Jane Cawley | Canada | Educational Consultant, PLEY program (Dalhousie University) |
| Janet Loebach | USA | Cornell University |
| Janet Omstead | Canada | Play for Life |
| Jarrett Kentzell | Canada | Hand in Hand Nature Education |
| Jasper Schipperijn | Denmark | World Playground Research Institute, University of Southern Denmark |
| Javier Brazo-Sayavera | Spain | University of Pablo Olavide, Sevilla |
| Joanne Josephidou | UK | Open University |
| John Weston | Canada | Canadian Health and Fitness Institute |
| Joy Wachira | Kenya | Kenyatta University |
| Julie Belair-Bak | Canada | ONP International |
| Justin Jeon | South Korea | Yonsei University |
| Justin Lang | Canada | Public Health Agency of Canada |
| Kamil Maciaszek | Poland | GratoSfera Foundation |
| Kathleen Ketchum | Canada | KKetchum Consulting, Wee Care and Casa Forest Licensed Child Care Agency |
| Kathy Hirsh-Pasek | USA | Temple University |
| Katie Hodder | Canada | Budding Minds |
| Kelly Arbour-Nicitopoulos | Canada | University of Toronto |
| Kevin Lamoureux | Canada | University of Winnipeg |
| Kimberly Hiscott | Canada | Andrew Fleck Children's Services |
| Kimberly Squires | Canada | University of Guelph |
| Kirsten Fons | Greenland | University of Greenland |
| Konstantina Rentzou | Greece | University of Ioannina |
| Kyoung June (David) Yi | Canada | McMaster University |
| Laurel Donison | Canada | Brock University |
| Lauren McNamara | Canada | Recess Project Canada |
| Lauren Turner | Canada | NorQuest College, Edmonton |
| Lisa Barnett | Australia | Deakin University |
| Lorena Morachimo | Italy | Francesco Tonucci |
| Louise Zimanyi | Canada | Humber College |
| Mads Bolling | Denmark | Bispebjerg and Frederiksberg Hospital |
| Mallory Donaldson | Canada | Outdoor Play Canada |
| Mariana Brussoni | Canada | UBC |
| Marina Delis | Canada | Lennoxville Elementary School |
| Marjaana Kangas | Finland | University of Lapland |
| Marjaana Kangas | Norway | Univeristy of Lapland |
| Mark Leather | UK | Plymouth Marjon University |
| Maxx Hartt | Canada | Queen's University |
| Meaghan Wilbur | Canada | Terry Fox Elementary, Anglophone North School District, New Brunswick |
| Megan Zeni | Canada | UBC |
| Melody Chan | Canada | Occupational Therapist, Vancouver Coastal Health |
| Michael Down | Australia | University of Notre Dame Australia |
| Michelle Bauer | Canada | BC Children's Hospital |
| Michelle Stone | Canada | Dalhousie University |
| Milos Zorica | Serbia | Serbian Preschool? |
| Narayan Subedi | Nepal | Nepal Development Society/Deakin University |
| Nevin Harper | Canada | University of Victoria |
| Nicola Kemp | UK | Canterbury Christ Church University |
| Nicola Maguire | Canada | Learning Enrichment Foundation |
| Odette Thevenot | Canada | Occupational Therapist at CHEO |
| Peter Bakalar | Slovakia | University of Prešov |
| Peter Bentsen | Denmark | Bispebjerg and Frederiksberg Hospital |
| Rachel Franz | USA | Fairplay for Kids |
| Rachel Jones | Australia | University of Wollongong |
| Rachel Ramsden | Canada | UBC |
| Rebecca Forte | Canada | RBC Canadian Open |
| Rebecca Seiling | Canada | CNAC |
| Richard Larouche | Canada | University of Lethbridge |
| Rita Cordovil | Portugal | University of Lisbon |
| Rob Ridley | Canada | Peel District School Board |
| Rob Wallis | Canada | Outward Bound Canada / Toronto & Region Conservation Authority (TRCA) |
| Ryan Fahey | Canada | Physical Health Education Canada |
| Salome Aubert | France | Active Healthy Kids Global Alliance |
| Sana Fakih | Canada | Child Health BC (CHBC) |
| Scott Mackenzie | New Zealand | Sport New Zealand |
| Shawnda Morrison | Slovenia, Singapore | National University of Singapore |
| Shelagh Pyper | Canada | Fuse Consulting |
| Sitong Chen | China | Shenzhen University |
| Son Truong | Canada | Dalhousie University |
| Soren Praestholm | Denmark | Copenhagen University |
| Steph N Dean | USA | George Mason University |
| Stephanie Prince Ware | Canada | Public Health Agency of Canada |
| Steven Lam | Kenya | International Livestock Research Institute |
| Susan Paudel | Nepal | Deakin University |
| Suzanne Levenson-Goldstein | USA | Merry Lea Environmental Learning Center |
| Tanya Halsall | Canada | The Royal Ottawa Hospital |
| Taru Manyanga | Canada, Zimbabwe | University of Northen British Columbia |
| Tim Gill | UK | Rethinking Childhood |
| Tracey Gage | Canada | Interpretive Guides Association |
| Tricia Rosengarten | USA | Merry Lea Environmental Learning Center |
| Trish Tucker | Canada | Western University |
| Trista Hollweck | Canada | University of Ottawa |
| Ulises Rodriguez | Canada | University of Lethbridge |
| Valerie Carson | Canada | University of Alberta |
| Wren Nicolardi | Canada | Rivers to Ridges |
| Yeong-Bae Kim | Canada | University of Alberta |
| YeonSoo Kim | South Korea | Seoul National University |
| Zach Rausch | USA | NYU-Stern School of Business |

**SUPPLEMENTAL FILE 2 – Environmental Scan to Inform the AOP10 Project Scope.**

Data from all relevant items were extracted into an Excel spreadsheet (Environmental Scan Items). Items were identified from: North America, South America, Europe, Africa, Asia, and Oceania. There were 80 items focused on outdoor play at the international level, and 56 items targeted outdoor play at the national level. Items were identified in a variety of languages when conducting the search strategy and were all translated – via Google Translate – into English or French for screening. There were 134 items in English and only 2 items in French. In terms of time periods, 97 items were from the 21^st^ century, whereas there were 36 from the 20^th^ century and only 3 from the 19^th^ century. There was a specifically high yield of items between the years 2020-2023 which generated 42 items. Overall, there was a variety of representation in outdoor play organizations/groups, but the majority of items were from the International Play Association (IPA), which accounted for 35 items.

Further information on extracted items is found in Tables A and B, below. We classified items based on their type (movements, formation of organizations, conferences, events, projects, and official documents) and their theme (right to play, nature and play, education and play, play structures, and children's play). The most common type of resource identified were conferences, such as ‘Breath of Fresh Air 2019 Outdoor Play Summit’, as seen in Table A. The least common resource type was movements, such as the ‘The Playground Movement’ (Table A). The most common theme of resources was ‘children’s play’ – a broad category that includes any item that aims to promote outdoor play – and the least common theme was ‘play structures’ (Table B).

**Table A. Summary of environmental scan results based on item type.**

| **Type** | **Example** | **Number of Items** |
| --- | --- | --- |
| Movements | The Playground Movement | 3 |
| Formation of organizations | Formation of Outdoor Play Canada | 23 |
| Conferences | Breath of Fresh Air 2019 Outdoor Play Summit | 42 |
| Events | National Play Outside Day | 21 |
| Projects | Legacy of Play Contest | 15 |
| Official documents | Position Statement on Active Outdoor Play | 32 |

**Table B. Summary of environmental scan results based on item theme.**

| **Theme** | **Example** | **Number of Items** |
| --- | --- | --- |
| Right to play | IPA Declaration of the Child’s Right to Play | 25 |
| Nature and play | Formation of Child and Nature Network | 8 |
| Education and play | 2023 Outdoor Learning Conference | 11 |
| Play structures | Public Playground Safety Handbook | 2 |
| Children's play | National Play Outside Day | 90 |

The results of the scan were also developed into a visual timeline on the Sutori platform ([Sutori Timeline (Environmental Scan)](https://www.sutori.com/en/story/environmental-scan-on-history-of-outdoor-play--GLEcuSQnQDpXWdUTakvhK6an); Sutori, Delaware, USA).

**SUPPLEMENTAL FILE 3 – Summary Statements Developed by ChatGPT to Inform the AOP10 Conceptual Framework**

| **Position Statements** | |
| --- | --- |
| Common/Main Themes | 1. **Importance of Play:** All the position statements emphasize the fundamental importance of play in children's lives. They recognize play as a child's right and acknowledge its significant role in healthy development, well-being, and learning.  2. **Benefits of Outdoor Play**: The statements highlight the numerous benefits of outdoor play, including physical health, mental well-being, social skills, creativity, and environmental awareness. Outdoor play is seen as essential for holistic child development.  3. **Equitable Access**: The position statements advocate for equitable access to play and outdoor learning opportunities for all children, regardless of their backgrounds, abilities, or geographical location.  4. **Inclusive Play Spaces**: The importance of creating inclusive and accessible play spaces is emphasized, ensuring that all children, including disabled children, can participate in play.  5. **Collaborative Decision-Making**: The statements call for collaborative decision-making processes that involve the perspectives of children, youth, educators, and various stakeholders in shaping policies and practices related to play and outdoor learning.  6. **Supporting Education and Health**: The position statements emphasize the positive impact of play and outdoor learning on academic performance, social development, physical health, mental well-being, and long-term health trajectories.  7. **Challenges and Barriers**: The statements identify and address barriers that limit children's access to play and outdoor learning, such as misconceptions about risks, overprotection, inadequate play spaces, financial limitations, and societal pressures.  8. **Recommendations for Action**: Each statement provides recommendations and calls for action from policymakers, educators, governments, communities, and various organizations to support and promote outdoor play and learning.  9. **Evidence-Based Approach**: Many of the statements rely on scientific evidence and research to support their claims about the benefits of play and outdoor learning.  10. **Recognition of Children's Rights**: Several position statements acknowledge and reference international conventions and treaties that recognize play as a fundamental right for children. |
| Overall Summary | *“Overall, these themes reflect a collective understanding of the importance of play in children's lives and the need to create supportive environments and policies that enable all children to engage in outdoor play and learning experiences. The statements also underscore the role of collaboration and evidence-based practices in promoting positive outcomes for children's development and well-being.”* |
| **Steering Committee Expressions of Interest – Content Themes** | |
| Common/Main Themes | **1. Expertise and Specialization:** A consistent theme is the demonstration of expertise, qualifications, and specialization in various relevant fields, showcasing the authors' readiness to contribute meaningfully.  **2. Commitment to Research and Scholarship:** The expressions consistently reflect a commitment to research and scholarly pursuits, highlighting the authors' engagement in academic endeavors, publications, and projects.  **3. Desire to Contribute:** All expressions of interest convey a strong desire to actively contribute to the specific initiatives or projects. The authors are enthusiastic about sharing their knowledge and skills.  **4. Passion for the Subject:** Passion for the subject matter, whether it's outdoor play, childhood development, or related fields, is evident across the board. Authors express genuine enthusiasm for their chosen areas of interest.  **5. Collaboration and Engagement:** A shared focus on collaboration, working in teams, and engaging with colleagues, stakeholders, and practitioners is evident in all the expressions.  **6. Impact and Contribution:** The authors consistently emphasize their intention to make a positive impact and contribute to positive changes within their respective fields.  **7. Holistic Perspective:** Many expressions of interest underscore the importance of a holistic perspective, considering diverse age groups, identities, and social equity in their contributions.  **8. Academic Background and Accomplishments:** Academic background, qualifications, and accomplishments are often highlighted to establish the authors' credibility and suitability for the projects.  **9. Global and Local Engagement:** Many authors express a global perspective, involving international collaborations or addressing local challenges with global implications.  **10. Leadership and Advocacy:** The expressions reflect authors' roles in leadership, advocacy efforts, and their willingness to take on responsibilities in their fields.  **11. Future Impact:** Several authors focus on the potential future impact of their contributions, whether it's influencing policy, advancing research, or fostering positive change. |
| Overall Summary | *“Overall, these common themes highlight the passion, expertise, commitment, collaboration, and potential impact of individuals who are interested in contributing to various projects and initiatives within their respective fields.”* |
| **Steering Committee Expressions of Interest – Areas of Expertise and Areas of Interest** | |
| Common/Main Themes – Areas of Expertise | 1. **Outdoor Play and Physical Activity:** Multiple individuals possess expertise in understanding and promoting outdoor play and physical activity, particularly in relation to children's development and well-being. 2. **Research Methodologies:** Qualitative research, survey design, data analysis, and synthesis review are common areas of expertise across several individuals, reflecting a shared proficiency in research methodologies. 3. **Health and Well-Being:** Expertise in promoting health, well-being, and positive health behaviors is a common thread among many individuals, with a focus on children and adolescents. 4. **Advocacy and Leadership:** Many individuals exhibit expertise in advocacy, policy development, and leadership roles within the realm of outdoor play, child development, and public health. 5. **Collaboration and Global Perspective:** Collaboration on national and international scales is a recurring theme, indicating an inclination towards global perspectives and cross-disciplinary teamwork. |
| Common/Main Themes – Areas of Interest | 1. **Equity and Inclusion:** Many individuals express an interest in promoting equitable access to outdoor play and physical activity opportunities, particularly for underserved, disabled, and marginalized populations. 2. **Physical Literacy:** Interest in promoting physical literacy—comprehensive physical, psychological, social, and cognitive development—is shared among several individuals. 3. **Community Engagement and Advocacy:** Interest in engaging communities, advocating for policy changes, and raising awareness about the benefits of outdoor play is a common area of focus. 4. **Nature-Based Education:** Several individuals are keen on nature-based education, exploring the benefits of nature engagement for children's holistic development. 5. **Child and Family-Centered Approaches:** The importance of incorporating children's and families' perspectives and needs into research, programs, and initiatives is a shared interest. 6. **Impactful Research and Translation:** Many individuals are interested in conducting research that has a positive impact on policy, practice, and community well-being, often emphasizing the translation of research findings into actionable strategies. 7. **Interdisciplinary Collaboration:** An interest in collaborating across disciplines and sectors to address complex issues related to outdoor play, child development, and public health is evident among multiple individuals. |
| Overall Summary | *“Multiple individuals share expertise in promoting outdoor play and physical activity for children's development and well-being. They possess skills in research methodologies, health promotion, and advocacy, often collaborating globally to advance equitable access, physical literacy, nature-based education, and child-centered approaches while translating impactful research across disciplines.”* |

**SUPPLEMENTAL FILE 4 – Text Mining Search Strategy**

| 1 | Active outdoor play | TS=((play* NEAR/5 outdoor) OR (time NEAR/5 outdoor) OR (outdoor NEAR/5 activit*) OR (nature NEAR/5 play)) |
| --- | --- | --- |
| 2 | One health | TS=(“one health”, interconnect* OR multidisciplinarity) |
| 3 | Climate change | TS=(“climate change” OR “climate crisis” OR “climate action” OR “climate justice” OR “renewable energy” OR “climate policy” OR “weather chang*” OR “planetary health” OR “ecological determinant*” OR (climate NEAR/5 (mitigat* OR adapt*)) OR prepare OR “hot day*” OR “cold day*” OR “temperature” OR “precipitation” OR “greenhouse gas* effect” OR “CO2” OR “O3” OR “N2O” OR “CH4” OR “air pollution” OR “PM 2.5” OR “PM 10” OR (rise NEAR/5 sea level*) OR “poor air quality” OR “natural disaster*” OR “extreme rain fall*” OR drought OR flood OR storm OR bushfire OR (substantial NEAR/5 temperature variability) OR “seasonality pattern*” OR “wet spring” OR “hot summer” OR “snowy winter” OR “barometric pressure” OR precipitation OR “extreme weather event*” OR “heat wave*” OR blizzard OR allerg* OR dust OR pollen OR “disease vector*” OR tick OR bug OR blackflies OR mites OR mosquitoes OR “global warming” OR biodiversity) |
| 4 | Mental health | TS= (mental* OR stress OR depression OR anxiety OR “PTSD” OR trauma OR eco-anxiety OR (radical NEAR/5 self-care) OR therap* OR “healing” OR “trauma-informed care” OR “coping” OR “emotional regulation” OR resiliency OR thriving OR flourishing OR confiden* OR “autonomy*” OR self-regulation) |
| 5 | Environmental stewardship | TS = (“environmental stewardship” OR (relationship NEAR/5 nature) OR “environmental awareness” OR “environmental conservation” OR “sustainability” OR “ecological system*” OR “resource management” OR (preservation NEAR/5 nature habitat*) OR “conservation” OR (nature NEAR/5 connection) OR “environmental health” OR “nature literacy”) |
| 6 | Crime | TS= (crime OR (crime NEAR/10 prevent*) OR (at-risk NEAR/5 youth) OR (neighborhood NEAR/5 crime), (neighborhood NEAR/5 safety) OR cohesion OR engagement OR surveillance OR community OR pro-social OR (conflict resolution) OR “stranger danger*” OR kidnap*) |
| 7 | Overall health and well-being | TS=(health OR wellbeing OR well-being OR growth OR development OR ageing OR problem-solving OR resilience OR teamwork OR leadership OR self-confidence OR “physical activity” OR exercise OR fitness OR “gross motor skill*” OR "social skill*” OR “emotional well*” OR “cognitive development” OR creativity OR imagination OR risk-taking OR risk* OR independence OR collaboration OR “sensory stimulation” OR “vitamin D” OR playfulness OR learning OR “inter-personal skil*” OR “physical literacy”) |
| 8 | Cultural appreciation & global understanding | TS= ((cultur* NEAR/5 appreciat*) OR (global NEAR/5 understand*) OR “cultural responsibility” OR “cultural accountability” OR “cultural safety” OR governance OR “cultural respect” OR reconciliation OR “social health”) |
| 9 | EDI | TS = (“EDI” OR “EDII” OR accessib* OR equit* OR divers* OR Inclusi* OR marginaliz* OR disadvantag* OR social justice OR inequality* OR gender OR rac* OR politic* OR ideolog* OR equity-deserving OR equity-seeking OR religio* OR muslim OR minorit*) |
| 10 | Parenting | TS= (“parenting” OR hyper-parenting OR bubble-wrapping OR tiger-parenting OR helicopter-parent OR “little emperor” OR “concerted cultivation”) |
| 11 | Other relevant areas/ field | TS= (“adventure education” OR (outdoor NEAR/5 education) OR (outdoor NEAR/5 learning) OR (outdoor NEAR/5 teaching) OR “outdoor pedagogy” OR “playground equipment” OR “playground surfacing” OR “benefit-risk assessment”) |

**SUPPLEMENTAL FILE 5 – Systematic Review Contributions Strategy**

|  | Activity | # of Contributors | Contributors | Progress Update |
| --- | --- | --- | --- | --- |
| 1 | Population, Intervention, Comparison, Outcome Framework | Lead author, student lead | All contributors to review |  |
| 2 | Registration | Lead author, student lead | All contributors to review |  |
| 3 | Conduct the search | Librarian, lead author, student lead |  |  |
| 4 | Develop the protocol | Lead author, student lead | All contributors to review |  |
| 5 | Screening - Level 1 (titles and abstracts) | 2-5 contributors |  |  |
| 6 | Screening - Level 2 (full text) | 2-5 contributors |  |  |
| 7 | Data Extraction | 2-5 contributors |  |  |
| 8 | Risk of Bias Assessment | 2-3 contributors |  |  |
| 9 | Data Synthesis | 2-5 contributors |  |  |
| 10 | Methods | *Completed by authors contributing to the above steps* | *Completed by authors contributing to the above steps* |  |
| 11 | Table: Summary of Papers | 1-5 contributors |  |  |
| 12 | GRADE Rating | 1-3 contributors |  |  |
| 13 | Manuscript writing | Lead author, student lead | All contributors to review |  |
| 14 | Consultation with knowledge translation stakeholders | 1-5 contributors |  |  |
| 15 | Review/Editing | All contributing authors | All contributors to review |  |
|  |  |  |  |  |
